# ESBL-Producing *Escherichia coli* Bacteremia: Association Between Virulence-Factor Burden, Source of Infection, and Clinical Features

**DOI:** 10.3390/pathogens15101086

**Published:** 2026-10-16

**Authors:** Tri Pudy Asmarawati, Wahyu Setyarini, Fikri Sasongko Widyatama, Hari Basuki Notobroto, Nasronudin Nasronudin, Toshiro Shirakawa, Kuntaman Kuntaman

**Affiliations:** 1 Doctoral Programme of Medical Science, Faculty of Medicine, Universitas Airlangga, Surabaya 60131, Indonesia; tpasmarawati@fk.unair.ac.id; 2 Department of Internal Medicine, Faculty of Medicine, Universitas Airlangga, Surabaya 60131, Indonesia; 3 Department of Internal Medicine, Universitas Airlangga Hospital, Surabaya 60115, Indonesia; 4 Institute of Tropical Diseases, Universitas Airlangga, Surabaya 60115, Indonesia; wahyu_setyarini82@yahoo.com (W.S.); fikri.sasongko@fk.unair.ac.id (F.S.W.); 5 Department of Medical Microbiology and Parasitology, Faculty of Medicine, Universitas Airlangga, Surabaya 60131, Indonesia; 6 Division of Biostatistics and Population Studies, Faculty of Public Health, Universitas Airlangga, Surabaya 60115, Indonesia; haribasuki.n@fkm.unair.ac.id; 7 Division of Advanced Medical Science, Kobe University Graduate School of Science, Technology and Innovation, Kobe 650-0017, Japan; toshiro@med.kobe-u.ac.jp; 8 Division of Urology, Department of Organ Therapeutics, Faculty of Medicine, Kobe University Graduate School of Medicine, Kobe 650-0017, Japan; 9 Department of Clinical Microbiology, Dr. Soetomo General Academic Hospital, Surabaya 60286, Indonesia

**Keywords:** *Escherichia coli*, extended-spectrum beta-lactamase, extraintestinal pathogenic *E. coli*, virulence factor, antimicrobial resistance

## Abstract

(1) Background: Extended-spectrum beta-lactamase (ESBL)-producing *Escherichia coli* are important causes of bacteremia, but integrated genotype–virulence–outcome data from low- and middle-income settings are limited; (2) Methods: Observational study of ESBL-producing *E. coli* (ESBL Ec) bacteremia at Dr. Soetomo General Academic Hospital, Surabaya, Indonesia (2022–2024). ESBL production was confirmed phenotypically and ESBL genes by PCR; eight ExPEC-associated virulence genes (*fimH*, *hlyA*, *iutA*, *cvaC*, *iha*, *traT*, *ompT*, *papC*) were detected by multiplex PCR and summed into a virulence-factor (VF) burden, classifying patients into low- and high-VF groups. (3) Results: Of 188 ESBL Ec isolates, 129 with complete clinical data were included. *bla*_CTX-M_ predominated (88.3%); *bla*_TEM_ and *bla*_SHV_ were less frequent. Isolates showed near-universal resistance to first- and third-generation cephalosporins, ampicillin, and ciprofloxacin, with relatively preserved susceptibility to carbapenems, amikacin, tigecycline, and cefoperazone/sulbactam. The most common virulence genes were *fimH* (81.9%), *traT* (78.7%), *iutA* (74.5%), and *ompT* (59.0%); overall mortality was 70.5%. High-VF isolates were more often from a urinary-tract source (77.6% vs. 59.2%; *p* = 0.026), whereas skin and soft-tissue and healthcare-associated infections predominated in the low-VF group. In a multivariable analysis, only *iha* remained independently associated with a urinary-tract source (adjusted odds ratio [aOR] 2.53, 95% CI 1.14–5.65). Independent predictors of mortality were the SOFA score (aOR 1.95 per point, 95% CI 1.48–2.56), male sex, and *traT* carriage. (4) Conclusions: ESBL Ec bacteremia in this setting is dominated by *bla*_CTX-M_ producers with extensive multidrug resistance and high mortality. Outcome was driven by illness severity at presentation rather than bacterial genotype. These findings are exploratory and require confirmation in larger multicenter studies with variant-level and genomic characterization.

## 1. Introduction

Extended-spectrum beta-lactamase (ESBL)-producing *Escherichia coli* has become a major cause of bloodstream infection worldwide, particularly in settings with high antibiotic use and limited access to novel agents [1,2]. These organisms are typically multidrug-resistant, frequently non-susceptible to third-generation cephalosporins and fluoroquinolones, and increasingly associated with carbapenem resistance, leaving few effective therapeutic options [3,4].

The global expansion of ESBL-producing *E. coli* has been driven predominantly by dissemination of the *bla*_CTX-M_ gene family. Since the early 2000s, *bla*_CTX-M_-type ESBLs have increasingly replaced *bla*_TEM_- and *bla*_SHV_-type ESBLs and are now the most common ESBL determinants among *E. coli* worldwide. Their dissemination is facilitated by plasmid-mediated horizontal gene transfer and the expansion of successful high-risk lineages, ST131 [5,6]. The clinical significance of *bla*_CTX-M_ extends beyond resistance to most cephalosporins, as it frequently correlates with resistance to fluoroquinolones and other antibiotic classes, which are often used to treat infections, especially in low- and middle-income countries.

Although *E. coli* commonly colonizes the human gastrointestinal tract, not all strains have the capacity to cause invasive infection. Distinguishing commensal strains from extraintestinal pathogenic *E. coli* (ExPEC) is therefore essential in bacteremia. Bloodstream infection results from an interaction between host susceptibility and bacterial pathogenic potential, rather than antimicrobial resistance alone. ExPEC is usually distinguished by the presence of virulence factors that distinguish it from commensal strains [7]. ExPEC possesses virulence-associated traits that support adhesion to host cells, epithelial invasion, iron acquisition in the bloodstream, serum resistance, immune evasion, and persistence within extraintestinal sites [7,8,9].

The characteristics and influence of these virulence factors on clinical manifestations and severity remain poorly understood [10,11]. We conducted a study of ESBL-producing *E. coli* bacteremia in adult patients to describe the resistance and virulence profile of ESBL-producing *E. coli* and to further explore the distribution of virulence-factor burden and their associations with host characteristics, clinical features, and infection outcomes. By providing local clinical and molecular epidemiological data, this study seeks to generate hypotheses regarding potential relationships among virulence-gene carriage, antimicrobial-resistance profiles, and clinical presentation.

## 2. Materials and Methods

This observational study was conducted among hospitalized patients with ESBL-producing *E. coli* bacteremia at Dr. Soetomo General Academic Hospital, Surabaya, Indonesia, between 2022 and 2024. This study continued the Tricycle Project for ESBL-producing *E. coli* surveillance in bacteremia, pregnant women, chickens, and the environment in Surabaya, Indonesia. Inclusion criteria were patients with a positive blood culture for *E. coli* and signs of infection during the study period. The presence of *E. coli* was confirmed by blood culture. Data on demography, comorbidities, potential sources of bacteremia, previous exposure, use of invasive medical equipment, and laboratory results were obtained from medical records.

### 2.1. Microbiology Test

Positive blood culture samples were detected using the BACTEC system. *E. coli* was identified using the BD Phoenix bacteriological identification machine (Becton Dickinson Phoenix M50 Automated Microbiology System, Sparks, MD, USA) with NMIC panels [12]. The production of ESBL was determined using a double-disk synergy test with ceftazidime, ceftazidime–clavulanic acid, and cefotaxime, as well as cefotaxime–clavulanic acid. Antibiotic resistance was interpreted according to the Clinical and Laboratory Standards Institute (CLSI) guidelines [13]. All ESBL-producing *E. coli* isolates were stored in Tryptic Soy Broth (TSB) with 15% sterile glycerol at −80 °C for further analysis.

### 2.2. DNA Extraction

ESBL-producing *E. coli* isolates obtained in this study underwent DNA extraction using the boiling method. Briefly, bacterial isolates were suspended in 400 µL of Tris-EDTA (TE) buffer, vortexed, and heated at 98 °C for 10 min. The supernatant was separated by centrifugation at 10,000 rpm for 10 min. The extracted DNA was subsequently stored at −20 °C until further amplification [14].

### 2.3. Multiplex PCR Reaction for bla_CTX-M_ and bla_SHV_ Genes and Singleplex PCR for bla_TEM_ and bla_OXA_ Genes

*E. coli* isolates were screened for the presence of ESBL-associated genes, including *bla*_CTX-M_, *bla*_SHV_, *bla*_TEM_, and *bla*_OXA_. The primer sequences and amplicon sizes for each ESBL gene are presented in Table S1. PCR amplification was performed using GoTaq^®^ Green Master Mix (Promega, Madison, WI, USA) in a final reaction volume of 25 µL. For multiplex PCR targeting *bla*_CTX-M_ and *bla*_SHV_, the reaction mixture consisted of 12.5 µL of GoTaq^®^ Green Master Mix, 5 µL of DNA template, 1 µL of each primer set, and 5.5 µL of nuclease-free water. The *bla*_TEM_ and *bla*_OXA_ genes were amplified separately by singleplex PCR using 12.5 µL of GoTaq^®^ Green Master Mix, 5 µL of DNA template, 2 µL of the respective primer set, and 5.5 µL of nuclease-free water.

For multiplex amplification of *bla*_CTX-M_ and *bla*_SHV,_ PCR cycling consisted of an initial denaturation at 95 °C for 6 min, followed by 35 cycles of denaturation at 95 °C for 1 min, annealing at 53 °C for 45 s, and extension at 72 °C for 1 min, with a final extension at 72 °C for 5 min. For singleplex amplification of *bla*_OXA_, PCR was performed with an initial denaturation at 96 °C for 5 min, followed by 35 cycles of denaturation at 96 °C for 1 min, annealing at 55 °C for 1 min, and extension at 72 °C for 2 min, followed by a final extension at 72 °C for 10 min. For singleplex amplification of *bla*_TEM_, PCR was performed with an initial denaturation at 96 °C for 5 min, followed by 35 cycles of denaturation at 96 °C for 1 min, annealing at 58 °C for 1 min, and extension at 72 °C for 1 min, followed by a final extension at 72 °C for 10 min. The PCR products were analyzed by electrophoresis on a 2% agarose gel. Positive controls for *bla*_CTX-M_, *bla*_SHV_, *bla*_TEM_, and *bla*_OXA_ were obtained from previously characterized bacterial isolates harboring the respective genes.

### 2.4. Phylogenetic Group Detection and Classification

The phylogroup of ESBL *E. coli* was determined using a modified Clermont method, involving multiplex quadruplex PCR and single PCR assay. Quadruplex PCR targeted four genes, namely arpA, chuA, yjaA, and TspE4.C2. The chuA, yjaA, and TspE4.C2 genes served as primary markers for phylogenetic grouping, while the arpA helped distinguish between phylogroups A and C. Additionally, all isolates underwent a single PCR targeting the *trpA* gene (219 bp) to further clarify classification, particularly differentiating phylogroups C, E, F, and cryptic clade I, according to Clermont’s algorithm. Table S2 presents the primers used. Phylogroup assignment was based on the presence or absence of amplification bands of each target gene. The PCR was performed in a total volume of 25 μL consisting of 2.5 μL 10× buffer, 0.2 mM each dNTP, 1 μL of DNA template (100 ng), 1.5 U Taq polymerase (Promega, Madison, WI, USA), primers at a concentration of 0.2 μM, and Milli-Q water to the final volume. The PCR for *arpA* included denaturation for 4 min at 94 °C, 30 cycles of 5 s at 94 °C and 20 s at 57 °C and a final extension step of 5 min at 72 °C. PCR for quadraplex and *trpA* included denaturation for 4 min at 94 °C, 30 cycles of 5 s at 94 °C and 20 s at 59 °C, and a final extension step of 5 min at 72 °C. PCR products were analyzed by 2% agarose gel electrophoresis, and DNA band visualization was based on the size of the amplification fragments.

### 2.5. Virulence Gene Detection of ESBL E. Coli

Genotypic identification of major virulence factors in ExPEC was performed using multiplex PCR. The selected genes were based on commonly identified virulence-associated genes, including hemolysins (*hlyA*), colicin V (*cvaC*), aerobactin (i*utA*), type 1 fimbrial adhesin (*fimH*), P-fimbriae (*papC*), serum resistance (*traT*), *iha,* and outer membrane protein (*ompT*) [14,15]. The eight genes were selected a priori to represent the principal functional categories of ExPEC pathogenesis for which validated, widely used primer sets are available and which have been most consistently reported in previous bacteremia and urinary-tract infection studies. The primer targets used for these virulence genes are listed in Table S3. The first primer pool consisted of *fimH*, *hlyA*, and *iutA*, whereas the second pool consisted of *cvaC*, *iha*, *ompT*, and *papC*.

PCR amplification was performed in a total reaction volume of 25 µL containing 1x standard Taq reaction buffer, 1.5 U Taq polymerase (New England Biolabs, Hitchin, UK), 0.2 µL each of forward and reverse primers, 0.2 nM dNTPs, 100 ng DNA template, and ultra-sterile water. The PCR cycling conditions for virulence-gene amplification pool consisted of *fimH*, *hlyA*, and *iutA* underwent denaturation at 94 °C for 3 min, followed by 29 cycles of denaturation at 94 °C for 1 min, annealing at 56 °C for 1 min, and a final extension at 72 °C for 10 min. Annealing of pool genes consisted of *cvaC*, *iha*, *traT*, *ompT*, and *papC* at 59 °C for 1 min. All amplicons were analyzed using agarose gel electrophoresis. For each isolate, a virulence-factor (VF) score was calculated as the number of virulence genes present and reported as low/high VF.

### 2.6. Demographic and Clinical Characteristics

Data on demography, comorbidities, sources of bacteremia, previous exposure, and use of invasive medical equipment were obtained from medical records. The comorbidities were calculated using the Charlson comorbidity index (CCI) [16]. Sources of bacteremia were identified retrospectively from clinical records based on signs and symptoms consistent with infection in the relevant organ system that were present before or at the onset of bacteremia. When available, source attribution was supported by microbiological evidence, including isolation of ESBL-producing *E. coli* from urine, sputum, wound/tissue, or other relevant clinical specimens. Previous exposure variables included previous hospitalization, use of an antibiotic, and an ICU stay of 30 days before the onset of bacteremia. The use of invasive devices was defined as the insertion of invasive mechanical ventilation, a CVC, or a urinary catheter for more than 48 h before the onset of bacteremia. Carbapenem-resistant *Enterobacterales* (CRE) are *Enterobacterales* that are resistant to at least one carbapenem. In this study, carbapenem resistance is reported throughout as carbapenem-resistant *E. coli.* The resistance breakpoints of carbapenems were determined according to CLSI M100 guidelines [13]. Healthcare-associated infection (HAI) was defined according to the National Healthcare Safety Network (NHSN) definition, in which a positive blood culture revealing *E. coli* was recorded on or after the third day of admission, with day of admission defined as calendar day 1 [17]. Severity of illness was defined as the SOFA score and the Pitt bacteremia score (PBS). Mortality was defined as in-hospital mortality.

Virulence-factor (VF) burden was defined as the total number of virulence-associated genes detected by PCR. To compare clinical characteristics, isolates were categorized as low- or high-VF burden based on the cohort median number of detected virulence factors. Isolates with a VF count equal to or below the median were classified as low-VF burden, while those above the median were classified as high VF burden. This median-based approach was used because no universally accepted clinical threshold exists for defining high-VF burden in this population. The discriminatory performance of the VF burden was assessed by receiver operating characteristic (ROC) curve analysis, and sensitivity analyses were performed across candidate cut-offs of four, five, and six genes (Table S5). Youden’s index was maximal at a cut-off of more than four genes, which coincides with the cohort median-based definition; therefore, we retained the median value of 4 as the primary cut-off. To address the possibility that the dichotomized score obscures the contribution of individual determinants, each of the eight genes was additionally compared individually between urinary-tract-source and non-urinary-tract-source bacteremia (Table S6).

### 2.7. Statistical Analysis

Data on resistance, virulence, demographics, and characteristics were presented as frequency and percentage for categorical variables. Numerical data were presented as the median (interquartile range, IQR). Normality of continuous data was assessed using the one-sample Kolmogorov–Smirnov test and obtained a result of *p* < 0.05. The total VF score was calculated and used to classify isolates into low VF and high VF burden groups, based on the median value. Comparisons between groups were made using the nonparametric Mann–Whitney test. Categorical variables were compared using the chi-square test. Because the comparison of low- versus high-VF-burden groups involved 28 tests, the family-wise error rate was substantial; we therefore also report the Bonferroni-corrected threshold (α = 0.05/28 = 0.0018) and Benjamini–Hochberg false-discovery-rate adjusted *q* values alongside the unadjusted *p*-values, and all comparisons are presented as exploratory rather than confirmatory. Molecular characteristics of included and excluded isolates were compared using the same tests (Table S4). Two multivariable binary logistic regression models with backward likelihood-ratio selection were fitted, one with urinary-tract-source bacteremia and one with in-hospital mortality as the dependent variable. Candidate covariates were chosen from variables with *p*-value < 0.25 in the bivariate analysis. For the urinary-tract-source model, these were the virulence-factor burden category, *iha*, *papC*, *hlyA*, sex, diabetes mellitus, carbapenem resistance, and CCI ≥ 3; for the mortality model they were *hlyA*, *traT*, *papC*, sex, hypertension, diabetes mellitus, pneumonia, intra-abdominal infection, urinary-tract source, carbapenem resistance, prior ICU stay, and the SOFA score. Model calibration was assessed with the Hosmer–Lemeshow goodness-of-fit test and discrimination with the Nagelkerke R^2^. Adjusted odds ratios (aOR) are reported with 95% confidence intervals. The data were analyzed using SPSS version 24 (Chicago, IL, USA; RRID: SCR_002865).

## 3. Results

In this study, 188 ESBL-producing *E. coli* isolates were examined for antimicrobial-resistance genes and virulence genes by multiplex PCR. We excluded 59 because the medical record data were incomplete, resulting in a total of 129 isolates that could be analyzed clinically and demographically.

### 3.1. ESBL Gene Patterns Among E. coli

**Figure 1:**
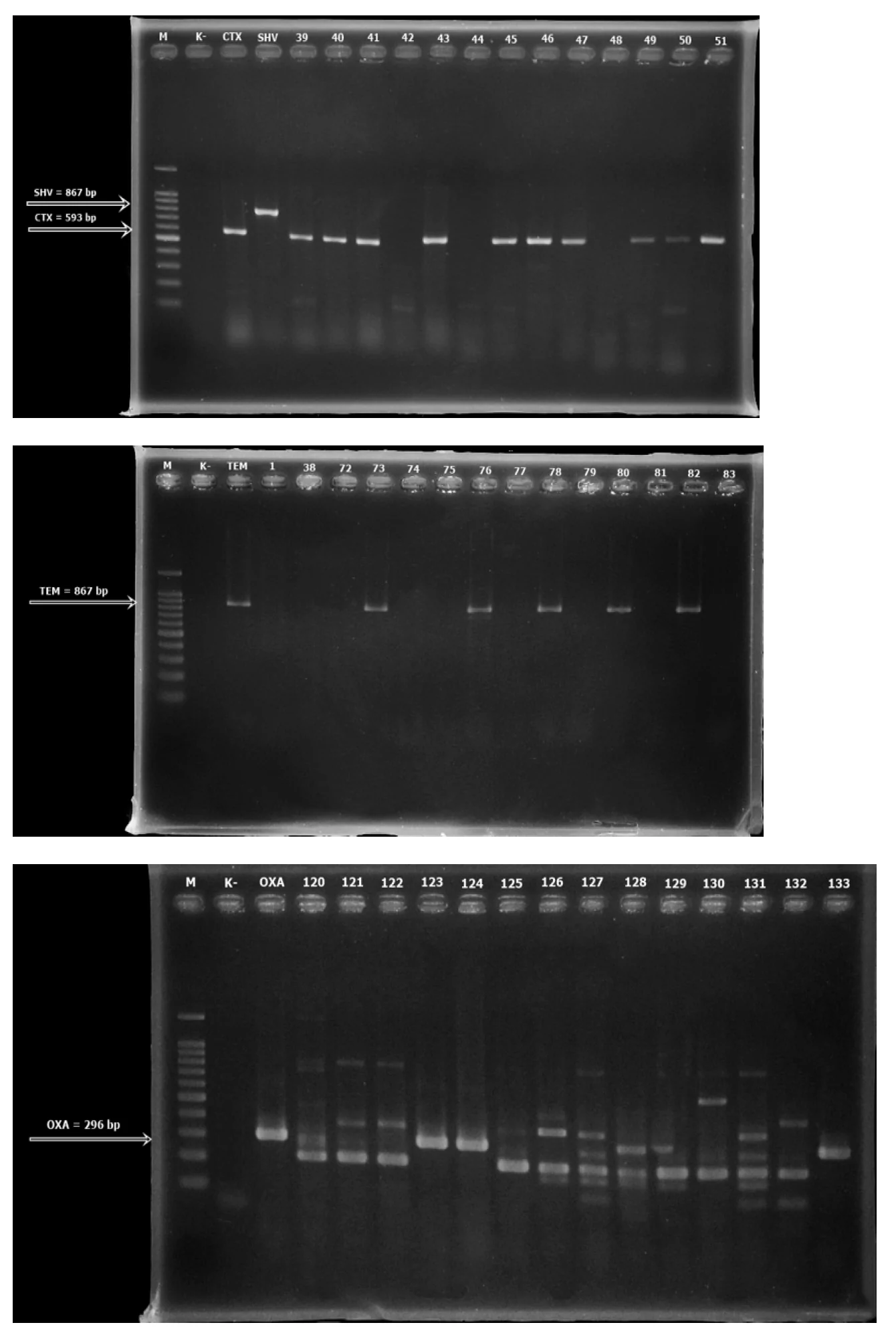
Agarose gel electrophoresis showed the *bla*_CTX-M_, *bla*_SHV_, *bla*_TEM_ and *bla*_OXA_ amplicons.

The distribution of ESBL genes in ESBL-producing *E. coli* isolates is shown in Table 1. The agarose gel electrophoresis showed the ESBL genes were displayed in Figure 1. In this study, the majority of isolates carried the *bla*_CTX-M_ gene, accounting for 166 (88.3%) of the total. The *bla*_TEM_ gene was detected in 55 (29.3%) isolates, and the *bla*_SHV_ gene was the rarest, detected in only six (3.2%) isolates. This pattern indicates that *bla*_CTX-M_ is the dominant determinant of ESBL genes in the ESBL *E. coli* population in this study.

### 3.2. Resistance Profile of ESBL E. coli

ESBL *E. coli* isolates exhibit very high resistance to first- and third-generation cephalosporins, with cefazolin resistance rates reaching 100% in all isolates and all ESBL gene variants (Table 2). Resistance to ampicillin was nearly 100% (99.5%), cefotaxime 99.5%, and ceftriaxone 97.8% in total isolates. Resistance to ceftazidime and cefepime was 74.9% and 93.1%, respectively, with OXA-producing isolates showing the highest resistance (91.9% and 100%).

Resistance to fluoroquinolones was very high, with ciprofloxacin resistance reaching 98.6% across all isolates and consistently high across all gene variants (97.1–98.4%). Levofloxacin and moxifloxacin showed resistance of 73.3% and 70.1%, respectively, with *bla*_OXA_ isolates having the highest resistance (91.9% and 86.5%). The combination of beta-lactams with beta-lactamase inhibitors, such as amoxicillin/clavulanic acid (84.4%) and piperacillin/tazobactam (84.6%), also showed high resistance, especially in *bla*_OXA_-producing isolates (97.1% and 94.6%).

Carbapenems maintained good activity with imipenem and meropenem resistance of only 21.2% and 17.6%, respectively. Amikacin showed the lowest resistance (2.1%) among all antibiotics tested. Tigecycline also has very low resistance (3.8%) and even 0% resistance in *bla*_OXA_ isolates, followed by cefoperazone/sulbactam (4.9%). Gentamicin showed 61.0% resistance across all isolates, with the highest resistance in *bla*_TEM_ isolates (78.2%). Resistance to trimethoprim–sulfamethoxazole was observed in 49.5% of isolates, with significant variation among the *bla*_CTX-M_ (49.4%), *bla*_TEM_ (61.1%), and *bla*_OXA_ (75.0%) genes.

### 3.3. Virulence Gene and Phylogenetic Classification in ESBL E. coli

**Figure 2:**
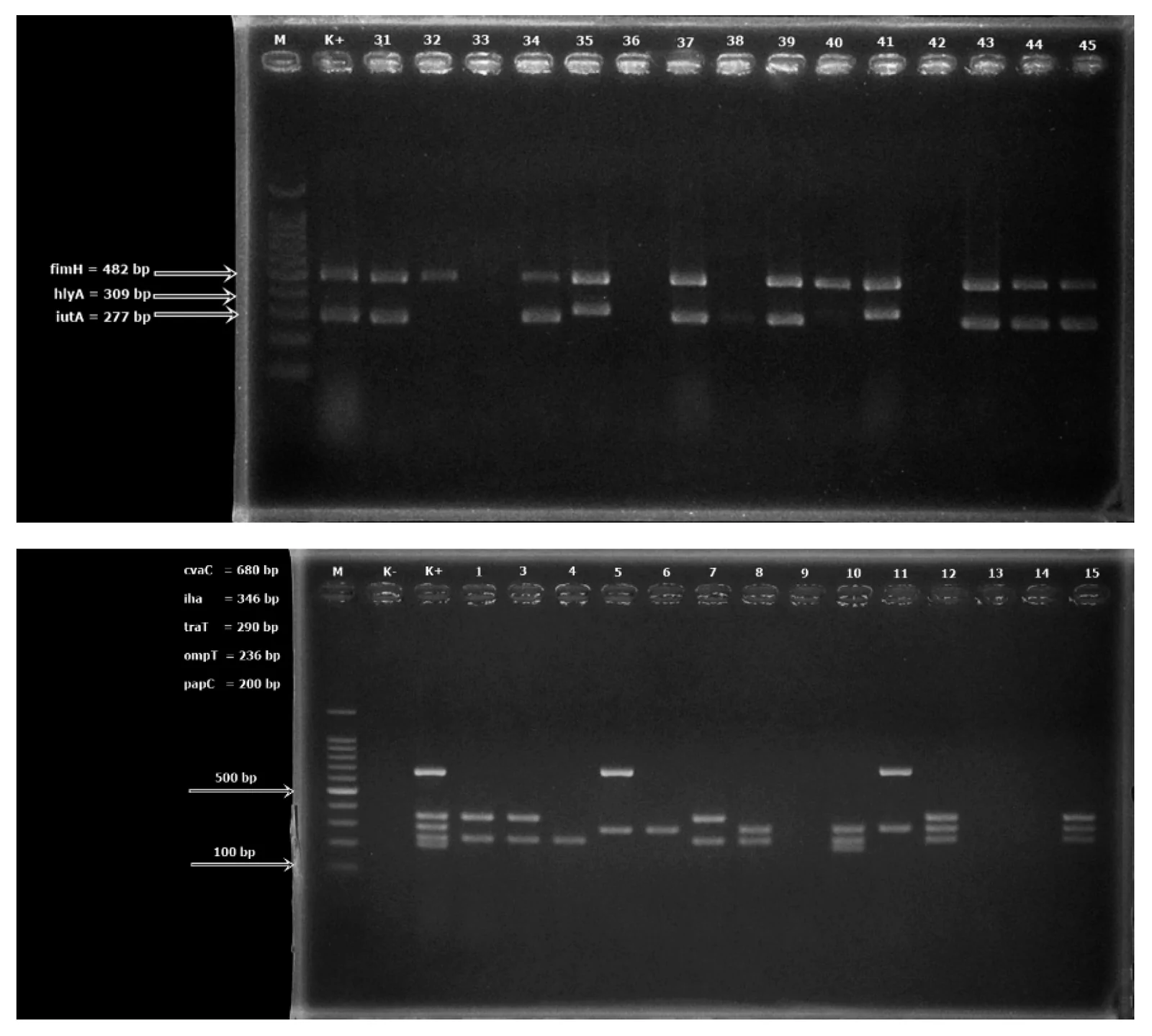
Agarose gel electrophoresis shows the *fimH*, *hlyA*, *iutA*, *cvaC*, *iha*, *traT*, *ompT*, and *papC* amplicons.

In this study, most ESBL *E. coli* isolates carried several key virulence genes (Table 3). The agarose gel electrophoresis showed the virulence genes displayed in Figure 2. The *fimH* adhesion gene was detected in 154 isolates (81.9%), whereas 34 isolates (18.1%) lacked it. The hemolysin *hlyA* gene was detected in only 16 isolates (8.5%). The siderophore *iutA* gene was found in 140 isolates (74.5%), while the colicin *cvaC* gene was identified in 38 isolates (20.2%). The adhesin gene was detected in 73 isolates (38.8%), while the *traT* gene, which confers resistance to serum, was detected in 148 isolates (78.7%). In addition, the *ompT* outer membrane protease gene was detected in 111 isolates (59.0%), and the *papC* pili gene was present in only 33 isolates (17.6%). This pattern suggests that *fimH*, *iutA*, *traT*, and *ompT* are the most commonly found virulence genes in ESBL *E. coli* isolates in this study.

**Figure 3:**
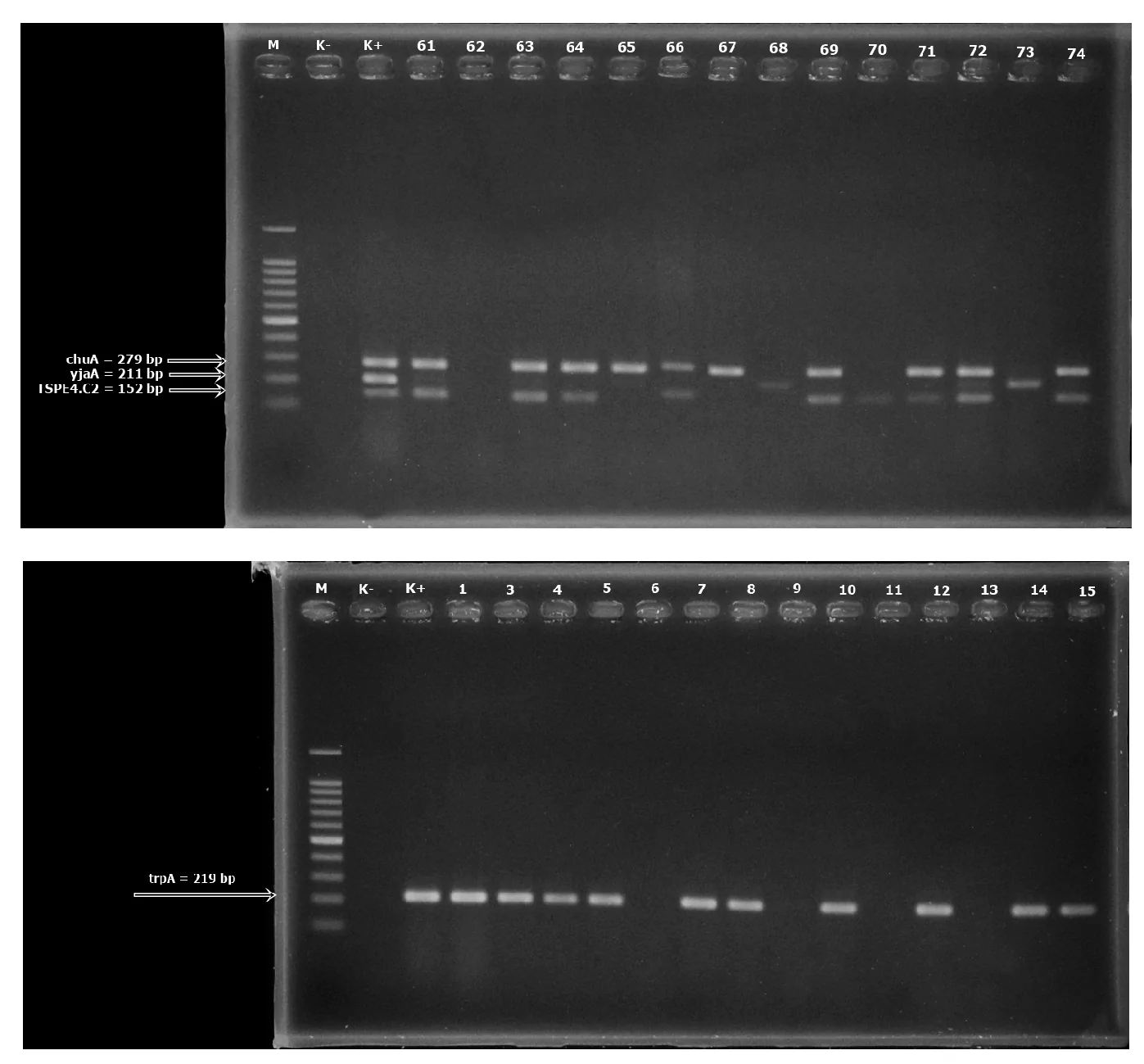
Agarose gel electrophoresis showed the *arpA*, *chuA*, *yjaA*, *TspE4.C2*, and *trpA* amplicons.

The phylogenetic classification *of* ESBL *E. coli* is shown in Table 4. The agarose gel electrophoresis showed the *arpA*, *chuA*, *yjaA*, *TspE4.C2*, and *trpA* amplicons displayed in Figure 3. In the isolates of this study, phylogroup B2 dominated the population, namely in 97 isolates (51.6%). Commensal phylogroups A and B1 were found in 11 (5.9%) and 13 (6.9%) isolates, respectively. Phylogroup D was found in 41 isolates (21.8%). The remaining 26 isolates (13.8%) could not be assigned to any of these groups by the Clermont algorithm and are reported as unassigned.

### 3.4. Bacteremia Subject Characteristics

Demographic characteristics showed a proportion of male subjects of 41.9% (54 patients). The median age of the study subjects was 54 years (IQR 43-63 years) (Table 5). Diabetes mellitus was the most frequent comorbidity with a prevalence of 39.5% (51 patients), followed by hypertension at 36.4% (47 patients). Charlson comorbidity index (CCI) ≥ 3 was found in 57.4% of subjects (74 patients). Pneumonia and urinary-tract infections (UTIs) were the most frequent sources of bacteremia; each was found in 67.4% of subjects (87). Skin and soft-tissue infections (SSTIs) were found in 24.0% of subjects (31), while intra-abdominal infections were found in 23.3% of subjects (30). The majority of patients (72.1%) had a history of previous hospitalization. More than half of the subjects (52.7%) had a history of previous antibiotic use. HAIs occurred in 45%. Carbapenem resistance was found in 12.5% of all ESBL *E. coli* isolates. The overall mortality rate reached 70.5% (91 patients). Survivors (*n* = 38) and non-survivors (*n* = 91) are compared in Table S7. Non-survivors were more often male (49.5% vs. 23.7%, *p* = 0.007) and less often hypertensive (29.7% vs. 52.6%, *p* = 0.014), and were markedly more severely ill at the onset of bacteremia, with a higher median SOFA score (6 [IQR 5–8] vs. 4 [IQR 2–5.25], *p* < 0.001), a higher median PBS (3 [IQR 1–6] vs. 0 [IQR 0–2], *p* < 0.001), and far more frequent high-care/ICU admission (72.5% vs. 18.4%, *p* < 0.001). Death occurred early: the median interval from the onset of bacteremia was 2 days (IQR 1–5) in non-survivors compared with a median of 9.5 days (IQR 6.75–13.25) to discharge in survivors (*p* < 0.001), and the overall length of stay was correspondingly shorter (6 vs. 13 days, *p* < 0.001). Among the virulence genes, only *hlyA* differed, being less frequent in non-survivors (4.4% vs. 23.7%, *p* < 0.001).

### 3.5. Clinical Comparison Based on the Virulence-Factor Burden

Bivariate analysis based on the number of virulence factors involved 129 patients divided into low VF (*n* = 71) and high VF (*n* = 58) groups, as shown in Table 5. HAIs were significantly more frequent among patients with low- rather than high-virulence-factor burden (53.5% vs. 34.5%, p=0.031). No statistically significant differences in virulence-factor-burden category were observed according to sex, age, comorbidities, CCI, CRE status, prior hospitalization, prior ICU stay, prior antibiotic use, mechanical ventilation, central venous catheterization, or urinary catheterization (all p>0.05).

In the bivariate analysis of clinical manifestations and outcomes, urinary-tract source of bacteremia was more common in the high-virulence-factor-burden group than in the low-burden group (77.6% vs. 59.2%, p=0.026). Conversely, skin and soft-tissue source was more frequent in the low-virulence-factor-burden group (31.0% vs. 15.5%, p=0.041). All *p*-values in Table 5 are unadjusted. Twenty-eight comparisons were performed, giving a Bonferroni-corrected significance threshold of *p* < 0.0018; none of the three nominally significant findings met this threshold (Bonferroni-adjusted *p* = 0.73 for urinary-tract source, 0.87 for healthcare-associated infection and 1.00 for skin and soft-tissue source), and the Benjamini–Hochberg false-discovery-rate adjusted *q* value was 0.268 for each. These findings should therefore be interpreted as exploratory signals rather than as established associations. Because a dichotomized composite score may obscure the contribution of individual determinants, each of the eight virulence genes was also compared between patients with urinary-tract-source bacteremia (*n* = 87) and those with a non-urinary source (*n* = 42) (Table S6). Only *iha* differed nominally between the two groups, being detected in 45 (51.7%) urinary-tract-source isolates versus 12 (28.6%) non-urinary isolates (*p* = 0.013). The remaining seven genes, including *papC* (25.3% vs. 11.9%, *p* = 0.080), *fimH* (82.8% vs. 81.0%, *p* = 0.802), *iutA* (79.3% vs. 71.4%, *p* = 0.321), *traT* (81.6% vs. 78.6%, *p* = 0.683), and *ompT* (66.7% vs. 59.5%, *p* = 0.427), were distributed similarly in both groups. High VF burden was present in 45 (51.7%) urinary-tract-source isolates versus 13 (31.0%) non-urinary isolates (*p* = 0.026). Applying the same correction as above to the eight-gene-level tests (Bonferroni threshold *p* < 0.0063), the *iha* association did not reach significance (Bonferroni-adjusted *p* = 0.104; Benjamini–Hochberg *q* = 0.104).

### 3.6. Multivariate Analysis for Urinary-Tract Source and In-Hospital Mortality in ESBL E. coli Bacteremia

In the model for urinary-tract-source bacteremia, the virulence-factor burden category was not retained (Table 6): it was removed at the second step and had not been significant in the full model (*p* = 0.483). The only virulence determinant that remained independently associated with a urinary-tract source was *iha* (aOR 2.53, 95% CI 1.14–5.65, *p* = 0.023), together with diabetes mellitus at borderline significance (aOR 2.24, 95% CI 0.99–5.11, *p* = 0.054). The final model was well calibrated (Hosmer–Lemeshow *p* = 0.958) but explained little of the variance (Nagelkerke R^2^ = 0.106) and classified cases no better than the null model (overall accuracy 67.4%, with none of the 42 non-urinary episodes correctly assigned).

In the model for in-hospital mortality, the SOFA score was by far the strongest independent predictor (aOR 1.95 per point, 95% CI 1.48–2.56, *p* < 0.001). Male sex (aOR 3.54, 95% CI 1.13–11.12, *p* = 0.030) and *traT* carriage (aOR 3.57, 95% CI 1.01–12.64, *p* = 0.048) were independently associated with death, whereas hypertension (aOR 0.18, 95% CI 0.06–0.54, *p* = 0.002) and *hlyA* carriage (aOR 0.16, 95% CI 0.03–0.82, *p* = 0.028) were inversely associated. This model discriminated well (Nagelkerke R^2^ = 0.557, overall classification accuracy 82.2%, correctly classifying 87.9% of non-survivors and 68.4% of survivors) and was well calibrated (Hosmer–Lemeshow *p* = 0.462).

## 4. Discussion

In this study, *bla*_CTX-M_ was the predominant ESBL determinant. This pattern parallels reports from many regions in which *bla*_CTX-M_-type enzymes have largely replaced earlier *bla*_TEM-_ and *bla*_SHV_-derived ESBLs as the dominant resistance mechanism in *E. coli* [18,19,20]. Phenotypically, the isolates showed almost universal resistance to first- and third-generation cephalosporins and ampicillin, very high resistance to fluoroquinolones, and substantial non-susceptibility to beta-lactam/beta-lactamase inhibitor combinations, consistent with the extensive multidrug-resistance profile typically described for *bla*_CTX-M_-producing ExPEC [18,21]. We highlighted high rates of resistance to ampicillin and third-generation cephalosporins because they remain clinically and epidemiologically informative markers of the resistance phenotype. ESBL enzymes hydrolyze most penicillins and cephalosporins, and reduced susceptibility to third-generation cephalosporins is a key phenotypic indicator of ESBL production. In our cohort, the predominance of *bla*_CTX-M_ provides a molecular explanation for the marked resistance to expanded-spectrum cephalosporins. Ampicillin resistance likewise reflects the limited activity of aminopenicillins against invasive *E. coli* isolates and the accumulated antimicrobial-selection pressure within the local setting [5]. For ESBL-producing *Enterobacterales* causing pyelonephritis or complicated urinary-tract infection, definitive therapy should be guided by susceptibility testing; carbapenems, fluoroquinolones, or trimethoprim–sulfamethoxazole may be appropriate options when active in vitro and clinically suitable [21,22].

An important feature of our data is that resistance extended well beyond the beta-lactam class, with ciprofloxacin resistance of 98.6%, tetracycline 75.9%, gentamicin 61.0%, and trimethoprim–sulfamethoxazole 49.5%. This co-resistance pattern is best understood as a consequence of the mobile genetic platforms that carry *bla*_CTX-M_ rather than of independent selection events. Characterization of CTX-M-encoding plasmids from extraintestinal *E. coli* has shown that most are transmissible, predominantly of IncF replicon type, flanked by IS*Ecp1*, and that recipient strains simultaneously acquire resistance to ciprofloxacin, trimethoprim–sulfamethoxazole, gentamicin, and amoxicillin–clavulanic acid together with the ESBL determinant; such plasmids also carry multiple addiction systems that favor their stable maintenance in daughter cells [23]. The particularly resistant phenotype of our *bla*_OXA_-positive subset, with ceftazidime resistance of 91.9%, cefepime 100%, levofloxacin 91.9%, and tetracycline 91.9%, would be compatible with *bla*_OXA-1_ being co-located on the same multireplicon element as *bla*_CTX-M_ rather than acting as an independent resistance driver [23]. The efficiency of such dissemination is underpinned by the biomechanics of the conjugative apparatus itself: the IncF-encoded F-pilus is simultaneously highly flexible and mechanically robust, and conjugation efficiency is maintained, or even enhanced, under hydrodynamic shear, conditions relevant to the urinary tract and bloodstream [24]. Because plasmid replicon typing and sequencing were not performed in the present study, this remains an inference rather than a demonstration in our isolates; nevertheless, it offers a coherent explanation for why a single dominant ESBL genotype was accompanied by such a uniformly broad resistance phenotype.

Therapeutically, carbapenems, amikacin (2.1% resistance), tigecycline (3.8%), and cefoperazone–sulbactam (4.9%) retained the best in vitro activity, whereas fosfomycin (28.9%) may retain a role in urinary-source infection. The very high piperacillin–tazobactam resistance (84.6%) is clinically salient given randomized evidence that piperacillin–tazobactam is not non-inferior to meropenem for ceftriaxone-non-susceptible *E. coli* and *Klebsiella pneumoniae* bloodstream infection [25]; in our setting, the in vitro data alone argue against its empirical use. Carbapenem resistance in 12.5% of the clinical cohort is a warning signal that this last-line option is already eroding locally. Polymyxins were not tested here, but it is relevant that colistin susceptibility in *Enterobacterales* depends on Lipid-A remodeling, in particular the 4-amino-L-arabinose modification of Lipid-A, which is under direct transcriptional control of envelope-stress signaling and can therefore shift with growth and stress conditions [26]. This argues for contemporaneous phenotypic testing rather than reliance on historical susceptibility data whenever polymyxins are considered.

The high proportion of B2 and D phylogenetic groups in ESBL *E. coli* consistent with previous reports shows that these phylogroups are frequently represented among ExPEC causing urinary tract and bloodstream infections [27,28] and closely resembles the proportion of phylogroup B2 reported for extraintestinal *E. coli* elsewhere [29]. In contrast, phylogroups A and B1 were less frequently identified. This distribution should be interpreted as a description of the phylogenetic composition of isolates in our cohort rather than evidence of the expansion of particular high-risk clones, because sequence typing, whole-genome sequencing, and clonality analyses were not performed [30,31]. It is well established that B2 and D lineages frequently carry adhesins, siderophore systems, toxins, and serum-resistance factors that favor the colonization of extraintestinal sites and tissue invasion [27,32,33]. We did not, however, analyze virulence-gene content by phylogroup, and our design cannot establish why these particular isolates caused invasive disease. The observation is therefore best stated descriptively: phylogroups typically associated with extraintestinal virulence predominated among bloodstream isolates in this cohort, a pattern compatible with, but not demonstrative of, a pathogenic advantage over the commensal-associated A and B1 lineages [34,35].

Most ESBL Ec isolates harbored adhesin and iron-uptake genes, particularly *fimH*, *iutA*, *traT*, and *ompT*, whereas *hlyA*, *cvaC*, *iha*, and *papC* were less frequent. This profile—strong adhesion, efficient iron uptake, and serum-complement resistance—is consistent with uropathogenic/extraintestinal pathogenic *E. coli* reported in other studies [10,28,36]. The *fimH* dominance is consistent with type 1 fimbriae being the most widely distributed chaperone-usher pathway (CUP) system in the *Enterobacteriaceae*, mediating mannose-sensitive binding to bladder uroplakins [37], consistent with the urinary tract being a leading source of bacteremia in our cohort. The lower frequency of *papC* and *hlyA* suggests that most isolates are not classically hypervirulent via P-fimbriae and hemolysin, but rely on general adhesion, plasmid factors, and serum-resistance mechanisms to maintain bacteremia [38]. This does not, however, imply an absence of adhesive capacity. *Pap* pili are controlled by a Dam-methylation/Lrp epigenetic switch and are repressed at lower temperatures, whereas type 1 fimbriae are governed by *FimB/FimE*-mediated inversion of *fimS*; in addition, the *Afa/Dra* and S-fimbrial families use homologous *PapI/PapB*-type regulators to achieve overlapping functions and were not included in our panel. Because CUP expression is phase-variable, PCR detection indicates capacity rather than expression—a caveat applying to every gene in our panel [37]. The high prevalence of *traT* and *ompT* may also have a plasmid dimension, since transfer of CTX-M-encoding plasmids confers serum resistance on recipient strains in most tested donor–recipient pairs while rarely conferring biofilm formation [29]; the detection of *cvaC*, a ColV plasmid-encoded marker, in one-fifth of isolates is consistent with this interpretation.

In our bivariate analysis comparing the burden of virulence factors, UTIs were significantly more common as a source of bacteremia in the high-virulence group, whereas SSTIs and HAIs were more frequent in the low-virulence group. This supports the linkage of virulence-gene enrichment, particularly adhesins and iron-uptake systems, to specific portals of entry rather than to overall comorbidity [36,39]. Ascending infection in a host with intact barriers requires intrinsic bacterial fitness, and urinary-adapted ExPEC lineages characteristically harbor adhesins, siderophore systems, and serum-resistance factors enabling ascent, invasion, and hematogenous spread [8,40,41]. Bacteremia originating from skin and soft tissue or from other healthcare-associated sources may therefore be caused by more opportunistic strains with fewer classical ExPEC virulence determinants but an enhanced ability to persist under antibiotic pressure and in device-related biofilms [23,42]. Notably, the relevant machinery is not captured by ExPEC panels: exopolysaccharide-dependent biofilm formation is governed by envelope-stress two-component signaling acting on the biosynthetic and transport operon promoters [26], and conjugative pili themselves mediate cell-to-cell aggregation and accelerate biofilm development independently of DNA transfer [24]. Biofilm phenotype was not assessed here; we regard it as the most plausible unmeasured explanation for the low-VF, device-associated phenotype. The difference between urinary- and non-urinary-source isolates was not driven by the most prevalent determinants: *fimH*, *iutA*, *traT*, and *ompT* were distributed almost identically. The signal came instead from the two least common adhesin-associated genes, *iha* (51.7% vs. 28.6%, *p* = 0.013) and, as a non-significant trend, *papC* (25.3% vs. 11.9%, *p* = 0.080). This is biologically coherent: *iha* encodes an adhesin and siderophore receptor and *papC* the usher of P-fimbriae, both implicated in urinary adherence and ascent, whereas type 1 fimbriae and aerobactin are near-ubiquitous in ExPEC and commensal *E. coli* alike. The bivariate association between VF burden and urinary-tract source therefore reflects a single adhesin rather than aggregate virulence-gene content, and the composite score adds nothing once *iha* is accounted for.

Higher VF burden was not associated with SOFA score, PBS, high-care/ICU admission, length of stay, or in-hospital mortality. This finding does not necessarily mean that virulence factors have no clinical relevance. First, the study may have lacked sufficient power to detect moderate differences after dividing the cohort into two relatively small groups, and dichotomizing a count variable at its median discards information and further reduces power. Second, as noted above, the presence of a gene does not guarantee its expression during infection, and interactions among virulence genes, phylogenetic background, bacterial lineage, and host factors were not evaluated. Third, and most probably, severity and mortality in *E. coli* bacteremia are influenced more strongly by host-related and process-of-care factors, including comorbidities, immune status, infection source and source control, severity at presentation, adequacy of antimicrobial treatment, and supportive care [9,43,44].

The in-hospital mortality of 70.5% observed here is considerably higher than the 10–20% typically reported for ESBL-producing *E. coli* bloodstream infection in high-income settings [1,44] and warrants explicit comment. This study was conducted in a national referral center, so the case mix is skewed toward critically ill and late-presenting patients. In addition, the near-universal resistance to cephalosporins and fluoroquinolones and the high piperacillin–tazobactam resistance mean that commonly used empirical regimens would frequently have been inactive, and inadequate empirical therapy is among the most consistent predictors of death in Gram-negative bacteremia [1,29,44]. Comparison of survivors and non-survivors (Table S7) supports this interpretation. Non-survivors were markedly more severely ill at the onset of bacteremia, whereas comorbidity burden, source of bacteremia, healthcare-associated acquisition, and prior exposures did not differ. Death occurred early, which leaves little opportunity for de-escalation or rescue once an inadequate empirical regimen has been started and points to the speed of clinical deterioration rather than to any property of the organism. Consistent with this, the SOFA score was the strongest independent predictor of death in multivariable analysis (aOR 1.95 per point, 95% CI 1.48–2.56), and neither carbapenem resistance nor the source of bacteremia remained in the model. Because treatment adequacy, time to appropriate therapy, and source control were not systematically abstracted, we cannot quantify their contribution, and this remains an important gap that future prospective work in this setting should close.

This study has several limitations. First, its single-center design and the 2022–2024 study period limit the generalizability of the local antimicrobial-resistance patterns, which may be influenced by institutional antimicrobial-use practices, infection-control measures, referral patterns, case mix, and circulating *E. coli* lineages. Second, ESBL genes were detected at the family level only and were not sequenced. Given that *bla*_CTX-M_ was carried by 88.3% of isolates, the absence of variant-level characterization is a major rather than a minor limitation: it prevents us from determining whether the local epidemiology is driven by CTX-M-15, CTX-M-27, CTX-M-14, or other variants, from relating variants to the ST131 sublineages with which they are globally associated, and from comparing our data directly with sequence-based surveillance elsewhere [20,29]. Third, 59 of 188 eligible isolates (31.4%) were excluded because clinical records were incomplete, which raises the possibility of selection bias. We compared the molecular characteristics of included and excluded isolates (Table S4); the analyzed subset was therefore not a random sample of all ESBL Ec bacteremia isolates and may be modestly enriched for CTX-M-producing, B2-lineage strains, which could inflate the apparent virulence-gene prevalence. Fourth, the predefined eight-gene panel did not capture the full virulence repertoire of ESBL Ec; each gene was weighted equally despite unequal biological contributions, and dichotomization at the cohort median reduces both statistical power and biological resolution. The low- and high-virulence-factor-burden categories should accordingly not be considered comprehensive or phenotypic measures of bacterial virulence, and they may not generalize to other populations or gene panels. Fifth, the multivariable models must themselves be interpreted cautiously. The models should be read as an adjustment check on the bivariate findings rather than as validated prediction models, and the individual adjusted odds ratios require replication in larger cohorts with pre-specified, non-stepwise model building. Sixth, we did not perform plasmid replicon typing, addiction-system profiling, or whole-genome sequencing, so we cannot determine whether the ESBL and virulence determinants observed here are physically linked on the same mobile element, as demonstrated in other settings [29]. Finally, the observational design precludes causal inference. Nevertheless, these findings provide contemporary local molecular and clinical data on ESBL *E. coli* bacteremia and generate hypotheses for larger, multicenter studies using broader genomic characterization and adjusted analyses.

Taken together, these results highlight the dual challenge posed by ESBL Ec bacteremia in our hospital: widespread resistance to commonly used empiric agents and the frequent presence of ExPEC virulence traits in urinary-source infections. Because the resistance phenotype observed here is most plausibly plasmid-associated, strategies directed at the mobile element, including limiting selection pressure through stewardship, interrupting conjugation, and targeting plasmid maintenance systems, may prove as valuable as new antibiotics [24,29,45]. Regulatory networks that simultaneously control virulence, biofilm formation, and envelope permeability are likewise attractive broad-spectrum targets, being highly conserved across Gram-negative pathogens [26,45]. In the immediate term, strengthening antimicrobial stewardship, optimizing empiric regimens for high-risk patients, and extending molecular surveillance to genomic characterization of circulating ExPEC lineages are the most actionable priorities in similar resource-limited settings.

## 5. Conclusions

ESBL *E. coli* bacteremia in this cohort was predominantly caused by *bla*_CTX-M_-producing, multidrug-resistant isolates and was associated with substantial mortality. Most isolates belonged to phylogenetic groups B2 and D and carried multiple virulence-associated genes. In-hospital mortality was predicted predominantly by severity of illness at presentation rather than by bacterial genotype, source of infection, or carbapenem resistance. These findings remain exploratory and require validation in larger multicenter studies with variant-level ESBL characterization, broader genomic profiling, and pre-specified multivariable model building. Nevertheless, the resistance burden and the very high mortality observed here highlight the importance of optimized empiric therapy, antimicrobial stewardship, and ongoing molecular surveillance.

## Figures and Tables

**Table 1:** ESBL gene distribution of ESBL *E. coli* (*n* = 188).

ESBL Gene	Positive	Negative
*bla*_CTX-M_	166 (88.3)	22 (11.7)
*bla*_TEM_	55 (29.3)	133 (70.7)
*bla*_SHV_	6 (3.2)	182 (96.8)
*bla*_OXA_	37 (19.7)	151 (80.3)

Note: ESBL = Extended-spectrum beta-lactamase.

**Table 2:** Patterns of antibiotic resistance in ESBL *E. coli* (*n* = 188).

Antibiotic Name	All Isolates (%)	*bla*_CTX_ (%)	*bla*_TEM_ (%)	*bla*_OXA_ (%)
Ampicillin	99.5	99.4	100	100
Cefazolin	100	100	100	100
Ceftazidime	74.9	73.3	77.8	91.9
Cefotaxime	99.5	99.4	98.2	100
Ceftriaxone	97.8	98.2	96.3	100
Cefoxitin	17.2	15.9	15.1	21.9
Cefepime	93.1	92.8	90.9	100
Imipenem	21.2	19.6	25.9	22.9
Meropenem	17.6	16.8	21.2	20.0
Ciprofloxacin	98.6	98.4	97.7	97.1
Levofloxacin	73.3	72.9	70.9	91.9
Moxifloxacin	70.1	69.8	69.8	86.5
Amikacin	2.1	2.4	3.6	8.1
Gentamycin	61.0	60.0	78.2	59.5
Amoxicilin–clavulanic acid	84.4	85.4	90.7	97.1
Piperacillin–tazobactam	84.6	84.9	90.9	94.6
Ampicillin–sulbactam	85.0	84.8	90.7	91.9
Cefoperazone–sulbactam	4.9	4.9	3.6	8.1
Trimetroprim–sulfamethoxazol	49.5	49.4	61.1	75.0
Fosfomycin	28.9	28.1	40	25
Aztreonam	91.4	92.1	90.7	100
Tigecycline	3.8	4.2	5.5	0
Tetracycline	75.9	75.8	83.6	91.9
Chloramphenicol	32.6	30.9	34.5	32.4

**Table 3:** Distribution of virulence genes in ESBL *E. coli* (*n* = 188).

Virulence Category	Virulence Gene	Positive *n* (%)	Negative *n* (%)
Adhesin	*fimH*	154 (81.9)	34 (18.1)
Adhesin, iron-acquisition	*iha*	73 (38.8)	115 (61.2)
Adhesin	*papC*	33 (17.6)	155 (82.4)
Toxin	*hlyA*	16 (8.5)	172 (91.5)
Iron-acquisition	*iutA*	140 (74.5)	48 (25.5)
Bacteriocin/plasmid-encoded factor	*cvaC*	38 (20.2)	150 (79.8)
Serum resistance (outer-membrane protein)	*traT*	148 (78.7)	40 (21.3)
Serum resistance (outer-membrane protein)	*ompT*	111 (59.0)	77 (41.0)

**Table 4:** Phylogenetic groups among ESBL *E. coli* (*n* = 188).

Group	*n*%
**A**	11 (5.9)
**B1**	13 (6.9)
**B2**	97 (51.6)
**D**	41 (21.8)
**Unassigned**	26 (13.8)

**Table 5:** Bivariate associations of low- versus high-virulence-factor burden among ESBL *E. coli* isolates (*n* = 129).

Variables	Total (*n* = 129)	Virulence-Factor (VF) Burden		*p*
		Low VF (*n* = 71)	High VF (*n* = 58)	
Demographic				
Male sex	54 (41.9)	27 (38.0)	27 (46.6)	0.329
Median age, years (IQR)	54 (43–63)	53 (38–62)	57 (48.75–64)	0.055
Comorbidities				
Hypertension	47 (36.4)	23 (32.4)	24 (41.4)	0.291
Diabetes mellitus	51 (39.5)	25 (35.2)	26 (44.8)	0.266
Heart failure	11 (8.5)	3 (4.2)	8 (13.8)	0.053
COPD	3 (2.3)	2 (2.8)	1 (1.7)	0.678
Liver cirrhosis	7 (5.4)	4 (5.6)	3 (5.2)	0.908
Hematologic malignancy	7 (5.4)	4 (5.7)	3 (5.2)	0.893
Solid tumor	28 (21.7)	17 (23.9)	11 (19.0)	0.495
CCI ≥ 3	74 (57.4)	40 (56.3)	34 (58.6)	0.794
Healthcare-associated infections	58 (45.0)	38 (53.5)	20 (34.5)	0.031 *
CRE	16 (12.5)	9 (12.9)	7 (12.1)	0.893
Previous exposure				
Prior hospitalization	93 (72.1)	50 (70.4)	43 (74.1)	0.640
Prior ICU stay	19 (14.7)	14 (19.7)	5 (8.6)	0.077
Prior antibiotic use	68 (52.7)	36 (50.7)	32 (55.2)	0.613
Use of invasive procedures or devices				
Mechanical ventilation	51 (39.5)	33 (46.5)	18 (31.0)	0.074
Central venous catheterization	78 (60.5)	46 (64.8)	32 (55.2)	0.266
Urinary catheterization	112 (86.8)	65 (91.5)	47 (81.0)	0.079
Source of bacteremia				
Pneumonia	87 (67.4)	51 (71.8)	36 (62.1)	0.239
Intra-abdominal	30 (23.3)	16 (22.5)	14 (24.1)	0.830
Urinary tract	87 (67.4)	42 (59.2)	45 (77.6)	0.026 *
Intracranial	4 (3.1)	2 (2.8)	2 (3.4)	0.837
Skin and soft tissue	31 (24.0)	22 (31.0)	9 (15.5)	0.041 *
Primary bacteremia	4 (3.1)	3 (4.2)	1 (1.7)	0.627
Median LOS (IQR)	8 (3–15)	8 (4–17)	6.5 (2–13.25)	0.086
Severity of illness at time of bacteremia				
High-care/ICU	73 (56.6)	44 (62.0)	29 (50.0)	0.172
Median SOFA score (IQR)	6 (4–7)	6 (4–7)	6 (4–8)	0.986
Median PBS (IQR)	2 (0-4)	2 (0-4)	2 (0-4)	0.253
Mortality	91 (70.5)	47 (66.2)	44 (75.9)	0.231

Note: * = significant at < 0.05. Data are expressed as n (%) unless otherwise stated. IQR: interquartile range; LOS: length of stay; COPD: chronic obstructive pulmonary disease; CCI: Charlson comorbidity index; CRE: carbapenem-resistant *Enterobacteriaceae*; ICU: intensive care unit; SOFA: sequential organ failure assessment; PBS: Pitt bacteremia score.

**Table 6:** Multivariable logistic regression models for urinary-tract-source bacteremia and for in-hospital mortality (*n* = 129).

**Panel A. Predictors of Urinary-Tract-Source Bacteremia.**			
**Variable**	**aOR**	**95% CI**	***p***
iha-positive	2.53	1.14–5.65	0.023
Diabetes mellitus	2.24	0.99–5.11	0.054
**Panel B. Predictors of in-hospital mortality.**			
**Variable**	**aOR**	**95% CI**	***p***
**SOFA score (per point)**	1.95	1.48–2.56	<0.001
**Hypertension**	0.18	0.06–0.54	0.002
**Male sex**	3.54	1.13–11.12	0.030
**traT-positive**	3.57	1.01–12.64	0.048
**hlyA-positive**	0.16	0.03–0.82	0.028
**Pneumonia**	2.60	0.82–8.26	0.105

Note: aOR, adjusted odds ratio; CI, confidence interval; SOFA, sequential organ failure assessment. Variables were selected by backward stepwise elimination based on the likelihood-ratio statistic; only variables retained in the final model are shown. Panel A: model chi-square 10.20, df 2, *p* = 0.006; Nagelkerke R^2^ 0.106; Hosmer–Lemeshow *p* = 0.958; overall classification accuracy 67.4%. Panel B: model chi-square 64.03, df 6, *p* < 0.001; Nagelkerke R^2^ 0.557; Hosmer–Lemeshow *p* = 0.462; overall classification accuracy 82.2%.

## Data Availability

The original data presented in this study are openly available at https://figshare.com/s/d2e0d8037383ab354da4 (accessed on 5 August 2026).

## Supplementary Materials

The following supporting information can be downloaded at https://www.mdpi.com/article/10.3390/pathogens15101086/s1, Table S1: Primer sequences and PCR product sizes of ESBL genes [46,47]; Table S2: Primer sequences and amplicon sizes used for phylogenetic analysis [48,49]; Table S3: Primer sequences for virulence-factor gene identification and their product sizes [14,50,51,52]; Table S4: Comparison of molecular characteristics between included and excluded ESBL *E. coli* isolates; Table S5: Sensitivity analysis of the virulence-factor burden cut-off; Table S6: Distribution of individual virulence genes and of virulence-factor burden according to the source of bacteremia in ESBL-producing *E. coli* (*n* = 129); Table S7: Comparison of survivors and non-survivors among patients with ESBL *E. coli* bacteremia (*n* = 129). References [14,46,47,48,49,50,51,52] are cited in the Supplementary Materials.

## Abbreviations

The following abbreviations are used in this manuscript:

BACTEC	Bacterial detection
BD	Becton, Dickinson and company
CCI	Charlson Comorbidity Index
CLSI	Clinical and Laboratory Standards Institute
COPD	Chronic obstructive pulmonary diseases
CRE	Carbapenem-resistance *Enterobacterales*
CTX-M	Cefotaximase-Munich
CUP	Chaperone-usher pathway
DNA	Deoxyribonucleic acid
dNTPs	Deoxyribonucleotide Triphosphates
*E. coli*	*Escherichia coli*
EDTA	Ethylenediaminetetraacetic Acid
ESBL	Extended-spectrum beta-lactamase
ExPEC	Extra-intestinal pathogenic *Escherichia coli*
HAIs	Healthcare-associated infections
ICU	Intensive Care Unit
IQR	Interquartile range
ITD	Institute of Tropical Disease
LOS	Length of stay
NMIC	Negative Microbial Identification Card
OXA	Oxacillinase
PBS	Pitt bacteremia score
PCR	Polymerase chain reaction
RPA	Recombinase Polymerase Amplification
SHV	Sulfhydryl variable
SOFA	Sequential organ failure assessment
SSTI	Skin and soft-tissue infection
TEM	Temoniera
TSB	Tryptic soy broth
UPEC	Uropathogenic *Escherichia coli*
UTI	Urinary-tract infection
VF	Virulence factor
