# Supplementary material for: ESBL-Producing Escherichia coli Bacteremia: Association Between Virulence-Factor Burden, Source of Infection, and Clinical Features
Source: Pathogens. 2026 Oct 16;15(10):1086. doi: 10.3390/pathogens15101086 (PMC13708686; doi:10.3390/pathogens15101086)
Supplement: Supplementary file 1 [file pathogens-15-01086-s001.zip › pathogens-4516144-supplementary.pdf]

Table S1. Primer sequences and PCR product sizes of ESBL genes

Gene	Primer name	Sequence 5′ to 3′	Product size	Reference
*bla*CTX-M	CTX-M-F	ATGTGCAGYACCAGTAARGT	593	[1]
	CTX-M-R	TGGGTRAARTARGTSACCAGA		
*bla*TEM	TEM-F	ATGAGTATTCAACATTTCCG	867	[1]
	TEM-R	CTGACAGTTACCAATGCTTA		
*bla*SHV	SHV-F	GGTTATGCGTTATATTCGCC	867	[1]
	SHV-R	TTAGCGTTGCCAGTGCTC		
*bla*OXA	OXA-F	ATTATCTACAGCAGCGCCAGTG	296	[2]
	OXA-R	TGCATCCACGTCTTTGGTG		

Table S2. Primer sequences and amplicon sizes used for phylogenetic analysis

Gene	Primer name	Sequence 5′ to 3′	Product size	Reference
chuA	chuA-F	ATGGTACCGGACGAACCAAC	288	[3]
	chuA-R	TGCCGCCAGTACCAAAGACA		[4]
yjaA	yjaA-F	CAAACGTGAAGTGTCAGGAG	211	[3]
	yjaA-R	AATGCGTTCCTCAACCTGTG		
TspE4.C2	TspE4.C2-F	CACTATTCGTAAGGTCATCC	152	
	TspE4.C2-R	AGTTTATCGCTGCGGGTCGC		
arpA	Acek-F	AACGCTATTCGCCAGCTTGC	400	
	arpA1-F	TCTCCCCATACCGTACGCTA		
trpA	trpAgpE-F	AGTTTTATGCCCAGTGCGAG	219	
	trpAgpE-R	TCTGCGCCGGTCACGCCC		

Table S3. Primer sequences for virulence factor gene identification and their product sizes

Gene	Primer name	Sequence 5′ to 3′	Product size	Reference
*fim*H	*fim*H-F	TCCCTACTACCAGCGAAAC	482	[5]
	*fim*H-R	CCCTACTGCTCCTAACGATAC		
*hly*A	*hly*A-F	CCAGAAGCAAGTCTTTGACC	309	[5]
	*hly*A-R	ACCTTATCATCCCCGTCTCCCAGG		
*iut*A	*iut*A-F	CTATAAAAGCCAGGGCGAC	277	[5]

Gene	Primer name	Sequence 5′ to 3′	Product size	Reference
	*iut*A-R	CTGCTGTGACGAAGAGAAAG		
*cva*C	*cva*C-F	CACACACAAACGGGAGCTGTT	680	[6]
	*cva*C-R	CTTCCCGCAGCATAGTTCCAT		
iha	*iha*-F	AACTGGCAGATCACCGAAGA	346	[7]
	iha-R	GCGACATCCAGTAATTTCGCT		
*tra*T	*tra*T-F	GGTGTGGTGCGATGAGCACAG	290	[6]
	*tra*T-R	CACGGTTCAGCCATCCCTGAG		
*pap*C	*pap*C-F	GTGGCAGTATGAGTAATGACCGTTA	200	[6]
	*pap*C-R	ATATCCTTTCTGCAGGGATGCAATA		
*omp*T	*omp*T-F	TTTGATGCCCCAGATATCTATCGG	236	[8]
	*omp*T-R	GGCTTTCCTGATATCCGGCCATG		

Table S4. Comparison of molecular characteristics between included and excluded ESBL *E. coli* isolates

	**Included cases (n=129)**	**Excluded cases (n=59)**	***p***
Virulence gene			
fimH	108 (83.7)	46 (78.0)	0.341
hlyA	11 (8.5)	5 (8.5)	0.990
iutA	100 (77.5)	40 (67.8)	0.156
cvaC	22 (17.1)	16 (27.1)	0.111
iha	55 (42.6)	18 (30.5)	0.113
traT	105 (81.4)	43 (72.9)	0.186
ompT	82 (63.6)	29 (48.2)	0.062
papC	26 (20.2)	7 (11.9)	0.166
ESBL gene			
bla_{CTX-M}	118 (91.5)	48 (81.4)	0.045
bla_{TEM}	35 (27.1)	20 (33.9)	0.344
bla_{SHV}	0 (0)	6 (10.2)	<0.001
bla_{OXA}	27 (20.9)	10 (16.9)	0.524
Phylogroup			
A	4 (3.1)	7 (11.9)	0.001

B1	9 (7.0)	4 (4.8)
B2	76 (58.9)	21 (35.6)
D	29 (22.5)	12 (20.3)
Unassigned	11 (8.5)	15 (25.4)

Table S5. Sensitivity analysis of the virulence-factor burden cut-off. Alternative cut-offs were derived from receiver operating characteristic (ROC) curve analysis with urinary-tract-source bacteremia as the outcome.

VF burden cut-off	Sensitivity (%)	Specificity (%)	Youden's index	Urinary-tract source (*n* = 87), *n* (%)	Non-urinary source (*n* = 42), *n* (%)	*p*
≥4 genes (>3.5)	70.1	42.9	0.130	61 (70.1)	24 (57.1)	0.145
≥5 genes (>4.5) – primary cut-off	**51.7**	**69.0**	**0.208**	**45 (51.7)**	**13 (31.0)**	**0.026***
≥6 genes (>5.5)	21.8	92.9	0.147	19 (21.8)	3 (7.1)	0.038*

The area under the ROC curve for virulence-factor burden as a predictor of urinary-tract-source bacteremia was 0.615 (95% CI 0.52–0.72; p = 0.025), indicating modest discrimination. Youden's index was maximal at a cut-off of >4 genes, which coincides with the cohort median-based cut-off used as the primary definition of high VF burden. The direction of the association was consistent across all three cut-offs. Comparisons were made with the Chi-square test; * = nominally significant at $p < 0.05$, unadjusted.

Table S6. Distribution of individual virulence genes and of virulence-factor burden according to the source of bacteremia in ESBL-producing *E. coli* (n = 129)

Virulence gene	Urinary-tract source (*n* = 87), *n* (%)	Non-urinary source (*n* = 42), *n* (%)	*p*
fimH	72 (82.8)	34 (81.0)	0.802
hlyA	11 (12.6)	2 (4.8)	0.163
iutA	69 (79.3)	30 (71.4)	0.321
cvaC	14 (16.1)	9 (21.4)	0.458
iha	45 (51.7)	12 (28.6)	0.013*
traT	71 (81.6)	33 (78.6)	0.683
ompT	58 (66.7)	25 (59.5)	0.427
papC	22 (25.3)	5 (11.9)	0.080
High VF burden	45 (51.7)	13 (31.0)	0.026*

Sources of bacteremia were not mutually exclusive; the urinary-tract group comprises all episodes in which a urinary focus was documented. All p-values are unadjusted; across the eight gene-level tests, the Bonferroni threshold is $p < 0.0063$, which the *iha* association does not meet (Bonferroni-adjusted p = 0.104; Benjamini–Hochberg q = 0.104).

Table S7. Comparison of survivors and non-survivors among patients with ESBL *E. coli* bacteremia (n = 129)

**Variable**	**Survivors (*n* = 38)**	**Non-survivors (*n* = 91)**	***p***
Male sex, *n* (%)	9 (23.7)	45 (49.5)	0.007*
Median age, years (IQR)	53 (42.75–61.50)	56 (40–64)	0.792
Median length of stay, days (IQR)	13 (9.75–21)	6 (2–11)	<0.001*
Median time after bacteremia onset, days (IQR)	9.5 (6.75–13.25)	2 (1–5)	<0.001*
Comorbidities			
Hypertension	20 (52.6)	27 (29.7)	0.014*
Diabetes mellitus	19 (50.0)	32 (35.2)	0.116
Heart failure	4 (10.5)	7 (7.7)	0.599
COPD	1 (2.6)	2 (2.2)	0.882
Liver cirrhosis	3 (7.9)	4 (4.4)	0.424
Hematologic malignancy	1 (2.7)	6 (6.6)	0.380
Solid tumor	7 (18.4)	21 (23.1)	0.559
CCI ≥3	22 (57.9)	52 (57.1)	0.937
Source of bacteremia			
Pneumonia	22 (57.9)	65 (71.4)	0.135
Intra-abdominal	5 (13.2)	25 (27.5)	0.079
Urinary tract	29 (76.3)	58 (63.7)	0.165
Intracranial	1 (2.6)	3 (3.3)	1.000
Skin and soft tissue	7 (18.4)	24 (26.4)	0.335
Primary bacteremia	3 (7.9)	1 (1.1)	0.076
Healthcare-associated infections	18 (47.4)	40 (44.0)	0.722
Carbapenem-resistant *E. coli*	2 (5.4)	14 (15.4)	0.122
Prior hospitalization	26 (68.4)	67 (73.6)	0.548
Prior ICU stay	3 (7.9)	16 (17.6)	0.157
Prior antibiotic use	18 (47.4)	50 (54.9)	0.432
Severity of illness at time of bacteremia			
Median Pitt bacteremia score (IQR)	0 (0–2)	3 (1–6)	<0.001*
Median SOFA score (IQR)	4 (2–5.25)	6 (5–8)	<0.001*
High-care/ICU admission	7 (18.4)	66 (72.5)	<0.001*
Virulence gene			
fimH	32 (84.2)	74 (81.3)	0.696
hlyA	9 (23.7)	4 (4.4)	<0.001*
iutA	28 (73.7)	71 (78.0)	0.595
cvaC	7 (18.4)	16 (17.6)	0.910
iha	17 (44.7)	40 (44.0)	0.935
traT	27 (71.1)	77 (84.6)	0.076
ompT	24 (63.2)	59 (64.8)	0.856

papC	12 (31.6)	15 (16.5)	0.055

Median time after bacteremia onset refers to the interval from bacteremia onset to hospital discharge in survivors and to death in non-survivors. * = nominally significant at $p < 0.05$. All p-values are unadjusted; 38 comparisons were performed, giving a Bonferroni-corrected threshold of $p < 0.0013$. CCI, Charlson Comorbidity Index; COPD, chronic obstructive pulmonary disease; SOFA, sequential organ failure assessment.

References

1. Ferreira, C.M.; Ferreira, W.A.; Almeida, N.C.O. da S.; Naveca, F.G.; das Graças Vale Barbosa Barbosa, M. Extended-Spectrum Beta-Lactamase-Producing Bacteria Isolated from Hematologic Patients in Manaus, State of Amazonas, Brazil. *Braz. J. Microbiol.* **2011**, *42*, 1076–1084, doi:10.1590/S1517-83822011000300028.
2. Kim, J.; Jeon, S.; Rhie, H.; Lee, B.; Park, M.; Lee, H.; Lee, J.; Kim, S. Rapid Detection of Extended Spectrum β-Lactamase (ESBL) for Enterobacteriaceae by Use of a Multiplex PCR-Based Method. *Infect. Chemother.* **2009**, *41*, 181–184, doi:10.3947/IC.2009.41.3.181.
3. Clermont, O.; Christenson, J.K.; Denamur, E.; Gordon, D.M. The Clermont Escherichia Coli Phylo-Typing Method Revisited: Improvement of Specificity and Detection of New Phylo-Groups. *Environ. Microbiol. Rep.* **2013**, *5*, 58–65, doi:10.1111/1758-2229.12019.
4. Clermont, O.; Bonacorsi, S.; Bingen, E. Rapid and Simple Determination of the Escherichia Coli Phylogenetic Group. *Appl. Environ. Microbiol.* **2000**, *66*, 4555, doi:10.1128/AEM.66.10.4555-4558.2000.
5. Ullah, N.; Assawakongkarat, T.; Akeda, Y.; Chaichanawongsaroj, N. Detection of Extended-Spectrum β-Lactamase-Producing Escherichia Coli Isolates by Isothermal Amplification and Association of Their Virulence Genes and Phylogroups with Extraintestinal Infection. *Scientific Reports 2023 13:1* **2023**, *13*, 1–13, doi:10.1038/s41598-023-39228-w.
6. Johnson, J.R.; Stell, A.L. Extended Virulence Genotypes of Escherichia Coli Strains from Patients with Urosepsis in Relation to Phylogeny and Host Compromise.
7. Colello, R.; Krüger, A.; Velez, M.V.; Del Canto, F.; Etcheverría, A.I.; Vidal, R.; Padola, N.L. Identification and Detection of Iha Subtypes in LEE-Negative Shiga Toxin-Producing Escherichia Coli (STEC) Strains Isolated from Humans, Cattle and Food. *Heliyon* **2019**, *5*, doi:10.1016/J.HELIYON.2019.E03015.
8. Desloges, I.; Taylor, J.A.; Leclerc, J.M.; Brannon, J.R.; Portt, A.; Spencer, J.D.; Dewar, K.; Marczynski, G.T.; Manges, A.; Gruenheid, S.; et al. Identification and Characterization of OmpT-like Proteases in Uropathogenic Escherichia Coli Clinical Isolates. *Microbiologyopen* **2019**, *8*, e915, doi:10.1002/MBO3.915.
